# Sfp-type PPTase inactivation promotes bacterial biofilm formation and ability to enhance wheat drought tolerance

**DOI:** 10.3389/fmicb.2015.00387

**Published:** 2015-05-21

**Authors:** Salme Timmusk, Seong-Bin Kim, Eviatar Nevo, Islam Abd El Daim, Bo Ek, Jonas Bergquist, Lawrence Behers

**Affiliations:** ^1^Department of Forest Mycology and Plant Pathology, Uppsala BioCenter, Swedish University of Agricultural SciencesUppsala, Sweden; ^2^Institute of Evolution, Haifa UniversityHaifa, Israel; ^3^Department of Physical and Analytical Chemistry, Uppsala UniversityUppsala, Sweden; ^4^Nova West Technologies & CommunicationsTucson, AZ, USA

**Keywords:** Sfp-type PPTase, *Paenibacillus polymyxa*, Evolution Canyon, rhizobacterial biofilm, plant drought tolerance, natural isolate genetic manipulation

## Abstract

*Paenibacillus polymyxa* is a common soil bacterium with broad range of practical applications. An important group of secondary metabolites in *P. polymyxa* are non-ribosomal peptide and polyketide derived metabolites (NRPs/PKs). Modular non-ribosomal peptide synthetases catalyze main steps in the biosynthesis of the complex secondary metabolites. Here we report on the inactivation of an A26 Sfp-type 4'-phosphopantetheinyl transferase (Sfp-type PPTase). The inactivation of the gene resulted in loss of NRPs/PKs production. In contrast to the former *Bacillus* spp. model the mutant strain compared to wild type showed greatly enhanced biofilm formation ability. A26Δ*sfp* biofilm promotion is directly mediated by NRPs/PKs, as exogenous addition of the wild type metabolite extracts restores its biofilm formation level. Wheat inoculation with bacteria that had lost their Sfp-type PPTase gene resulted in two times higher plant survival and about three times increased biomass under severe drought stress compared to wild type. Challenges with *P. polymyxa* genetic manipulation are discussed.

## Introduction

The global agricultural system is experiencing profound changes as a result of anthropogenic pressures. The ever-increasing human population (more than 9 billion by 2050), together with the impacts of climate change, is shaping what we eat, and how much, more than ever. A key challenge for plant growth is global water shortage, limiting crop yields already today in more than 70% (Foley et al., 2011; Smol, 2012). Against this background, the global food system will have to improve its resource use efficiency and environmental performance significantly to ensure the sustainability of global food production and consumption. The microbial world is a large unexplored reservoir of biodiversity which exists at diverse and sometimes extreme ecological niches. *Paenibacillus polymyxa* is a bacterium widely used in agriculture, industry, and environmental remediation because it has multiple functions (Choi et al., 2009; Timmusk et al., 2009, 2014; Timmusk and Nevo, 2011). Recently it was shown that the species has very high metabolic diversity which results in great differences in the bacterial potential for bio-technological applications (Timmusk et al., 2014). *P. polymyxa* strains from the harsh South Facing Slope (SFS) in comparison to the moderate North Facing Slope (NFS) at “Evolution Canyon,” Israel show huge differences in their metabolism, drought tolerance enhancement and biocontrol ability (Timmusk et al., 2005, 2011; Kim and Timmusk, 2013). Our *P. polymyxa* strain A26 is isolated from the stressful SFS and has been shown capable of moderate drought stress tolerance enhancement (Timmusk et al., 2011, 2014).

An important pool of the bioactive compounds of great interest for biotechnology are non-ribosomal peptides/polyketides (NRPs/PKs). The spectrum of application of both classes of compounds is large. NRPs are produced by non-ribosomal peptide synthetases (NRPS) and PKs by polyketide synthetases (PKS). Both are very diverse families of natural products with an extremely broad range of biological activities that include adaptation to unfavorable environments, and communication or competition with other microorganism in their natural habitat (Lambalot et al., 1996; Chen et al., 2009; Sunbul et al., 2009b; Powell et al., 2007; Timmusk and Nevo, 2011). The diverse structure of the compounds can be explained by how the NRPs and PKs molecules are synthesized. They are produced as secondary metabolites by microorganisms, by the consecutive condensation of amino acids. The process is not limited to the 20 protein amino acids. Around 500 different monomers, including monoproteinogenic amino acids, fatty acids, and α-hydroxy acids, have been identified as building blocks for NRPs and PKs (de Bruijn et al., 2007). The building blocks contribute to the structural versatility of the compounds and are likely to contribute substantially to the observed biological effect. Despite the enormous chemical diversity in non-ribosomal peptides/polyketides, NRPS and PKS and hybrid PKS-NRPSs share a common point of regulation (Sunbul et al., 2009a,b). These enzymes require activation by phosphopantetheinyl transferases (PPTases) (Beld et al., 2014; Bunet et al., 2014). Bacterial PPTases are divided into two groups based on their sequence homologies and substrate spectra. The members of the first group are associated with primary metabolism and catalyze the activation of the fatty acid acyl carrier domains (Beld et al., 2014; Bunet et al., 2014). The second group prototype is a PPTase Sfp which activates peptidyl carrier protein domains in *Bacillus subtilis* (Quadri et al., 1998; Beld et al., 2014; Bunet et al., 2014). This group of enzymes has been shown to exhibit broad spectrum activity. During the compound assembly, the biosynthesis intermediates are attached to carrier protein domains of these megaenzymes via a phosphopantetheinyl arm. The Sfp-type PPTases transfer 4′-phosphopantetheinyl (Ppant) groups from CoA to conserved serine residues on peptidyl carrier protein (PCP) and acyl carrier protein (ACP) domains (Sunbul et al., 2009a,b; Beld et al., 2014; Bunet et al., 2014). The post-translational modification of the PCP and ACP domains with Ppant as catalyzed by Sfp-type 4′-phosphopantetheinyl transferase is crucial for the activation of NRPS and PKS (Sunbul et al., 2009a,b; Beld et al., 2014; Bunet et al., 2014). The virulence of various pathogens is dependent on non-ribosomal peptide or polyketide production and their Sfp-type PPTases represent gate keepers to pathogenicity. Hence recently the enzyme has been highlighted as a potential target for antibacterial drug development in the medical industry as well as a means of fighting agricultural pathogens (Chalut et al., 2006; Leblanc et al., 2012; Zheng and Burr, 2013). Inhibition of the enzyme has been shown to reduce the pathogen's growth and virulence (Chalut et al., 2006; Leblanc et al., 2012; Zheng and Burr, 2013; Beld et al., 2014; Foley et al., 2014).

In the frame of the current work, a *P. polymyxa* A26 mutant with an inactivated Sfp-type PPTase gene (A26Δ*sfp*) was created. The mutant, which is incapable of producing the enzymatically active 4′-phosphopantetheinyl transferase, in turn results in a *P. polymyxa* A26 mutant strain lacking enzymatically active NRPS and PKS. In contrast to earlier reports on bacterial reduced growth caused by an Sfp-type PPTase mutation, A26Δ*sfp* was significantly enhanced in biofilm production. The A26Δ*sfp* strain had the effect of increasing plant dry weight during normal watering conditions and in particular when the plant was exposed to drought stress. To our knowledge the fact that a bacterial Sfp-type PPTase mutation can promote biofilm formation and enhance the benefits of bacterial interaction with eukaryotes has not been reported earlier.

## Materials and methods

### Bacterial strains

*P. polymyxa* A26 was isolated from the “Evolution Canyon” SFS as earlier described (Timmusk et al., 2011). *P. polymyxa* E681 was provided by SH Park (Ryu et al., 2005). *Bacillus subtilis* 3610 and *B. subtilis sfp*::mls (DS3337) were provided by DB Kearns (McLoon et al., 2011). The primers for this experiment are described in Table S1. *Escherichia coli* DH5alpha cells (Invitrogen) acted as host for recombinant plasmids and were cultivated at 37°C on LB agar. *P. polymyxa* A26 and E681 were grown in half strength Tryptic Soy Broth (TSB) (Difco) at 30°C. Brain Heart Infusion medium (Difco) containing 10% sucrose was used for the transformation of *P. polymyxa* A26 and E681. The extraction of plasmid and chromosomal DNA was performed according to standard procedures (Harwood and Cutting, 1990). For antibiotic selection the media were supplemented with 5 μg/ml chloramphenicol (Cm), 1 μg/ml ampicillin (Ap), final concentration.

### Construction of the suicide vector and inactivation of A26 Sfp-type PPTase gene

**Figure 1:**
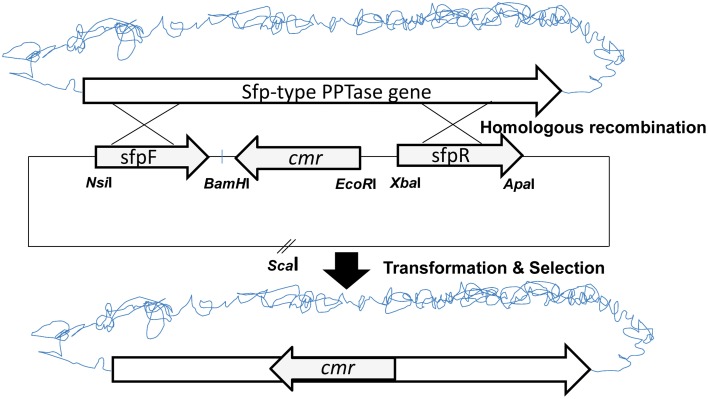
**Schematic drawing of the shuttle plasmid pGEM7Z-*sfp* and the inactivation strategy**.

To analyse the role of A26 Sfp-type PPTase in A26 non-ribosomal peptide production, the Sfp-type PPTase gene was inactivated (Figure 1) (Datsenko and Wanner, 2000; Kim and Timmusk, 2013). As the gene homologous recombination was not successful in A26 the initial gene replacement was performed in the *P. polymyxa* E681 strain. The gene replacement vector was constructed by cloning a chloramphenicol resistance marker gene (*cmr*) into the pGEM7Z/Cm *EcoR*I-*BamH*I site. Then the flanking regions of the Sfp-type PPTase gene in A26 were amplified by PCR from the A26 chromosomal DNA using the sfp FF and sfp FR primers or the sfp RF and sfp RR primers resulting in upstream and downstream fragments. The upstream fragment was cloned into the pGEM7Z/Cm vector at the *Nsi*I/*BamH*I site and the downstream fragment at the *Xba*I/*Apa*I restriction site (Figure 1). The gene replacement pGEM7Z/Cm-*sfp* plasmid was introduced into E681. Homologous recombination was confirmed by PCR and sequencing and resulted in the replacement of E681 Sfp-type PPTase gene. Then the E68Δ*sfp* chromosomal DNA was extracted and transferred to A26. The PCR and sequencing conformation analysis was repeated on genomic DNA extracted from A26Δ*sfp*.

### Transformation conditions for *P. polymyxa* E681 and A26

Transformation of *P. polymyxa* was performed by electroporation. In order to prepare competent cells, a single *P. polymyxa* E681 or A26 colony was picked from 1/2 TSA solid medium and cultured in BHIS broth (Harwood and Cutting, 1990) at 30°C and 200 rpm for 15 h. Two milliliters of the pre-culture was inoculated into 200 mL of BHIS and cultured at 30°C, 200 rpm. When the cells reached an *OD*_600_ of 0.5, the bacterial growth was interrupted by placing the culture on ice for 10 min and centrifuged at 5000 × *g* for 10 min at 4°C. After washing the cells twice with cold SM buffer (Harwood and Cutting, 1990), the competent cells were resuspended in SM buffer. Electroporation was performed using a Gene Pulser (Bio-Rad Laboratories, Richmond, Calif.) as described earlier (Kim and Timmusk, 2013). For *P. polymyxa* A26 transformation, 20 μg of chromosomal DNA was used instead of plasmid. Transformants were selected on Cm selection plates and cassette insertion was confirmed by PCR and sequencing.

### Complementation

For complementation the Sfp-type PPTase gene was amplified from A26 using primers A26 sfp LF and A26 sfp LR. The PCR product was purified, cleaved with EcoRI and BamHI, and cloned into pHPS9 (BGSC, Ohio), an *E. coli*/*Bacillus* shuttle vector, via the NdeI and BamHI restriction sites. The product, i.e. the pHPS9-*sfp* vector, was transformed into *E. coli*, purified using QIAprep Spin Miniprep Kit (Qiagen) confirmed by PCR and sequencing and finally transformed into A26Δ*sfp* as described above. The transformation resulted in complemented mutant strain (A26Δ*sfp*/pHPS9-*sfp*).

### Metabolic analysis

Carbohydrate metabolism shown to be characteristic for *P. polymyxa* species was studied using the BioMerieux API50CH system following the instructions provided by the manufacturer. Additional biochemical identification tests were performed using the BioMerieux API20E system following the instructions provided by the manufacturer.

### Bioassays for antibacterial and antifungal activity

The antagonistic ability of *P. polymyxa* A26 wild type, mutant and complemented strains was detected against *Fusarium graminearum* on potato dextrose agar (PDA) plates for 3 days at 30°C. A26 and A26Δ*sfp* lipopeptide extraction was performed as described by Li et al. (2007). Fusaricidin and polymyxin bioactivity assays have been previously described (Choi et al., 2008, 2009).

### Fusaridin and polymyxin detection by LC-MS and MALDI-TOF MS

Metabolite extraction and antimicrobial activity analysis for LC-MS was conducted as previously described by Choi et al. (2008). The final methanol extract was evaporated and dissolved in 2 ml water. The concentrated samples were further purified on a C18 column (Sili-Cycle Inc. Quebeck, CA) and eluted with a water/methanol gradient. Active eluted fractions were used for LC-MS. LC-MS was performed using a high pressure liquid chromatography system (Thermo Electron Co, USA). The samples were injected into reverse phase columns and analyzed in a mixed solvent of water and acetonitrile containing 0.1% formic acid (0.2 ml/min).

Lipopeptide extraction for MALDI-TOF MS was performed as previously described by Li et al. (2007). Bruker Ultraflex II mass spectrometer using alpha-hydroxycinnamic acid (alpha-HCCA) was used as matrix and spectra were visualized with the mMass software (Niedermeyer and Strohalm, 2012).

### Plant growth analysis

Winter wheat (cv. Stava) seeds were sterilized with 5% chlorine solution. A26 and A26Δ*sfp* strains were grown in TSB at 28°C overnight. Culture density was confirmed by colony forming analysis. Inoculation was performed by watering 7 day old plantlets with bacterial solutions containing 10^7^ bacteria. Wheat seeds were grown in pots filled with sand mixed with 10% greenhouse potting mix soil in a growth cabinet at 24/16°C (day/night) temperature, and 16 h photoperiod at 250 μmol m^2^ s^−1^. The soil moisture was adjusted to 75% of water holding capacity. Soil moisture (12.5% of soil dry weight) was kept constant during the first 10 days of seedling growth. In 10 days after germination drought stress was induced by stopping watering. Soil volumetric water content was evaluated using 5TE soil moisture sensors (Decagon Devices, Inc.). Root and shoot dry weights were determined after 14 days of drought stress. Plant survival was calculated daily after stress application using 32 stressed plants that were randomly selected and divided into two groups with 16 plants each. Plants were watered and allowed to recover for 4 days. The plants recovering were counted as survived plants. The survived and recovered plants were harvested, washed and dried. For estimates of roots with adhering soil, 12 plantlets per treatment were sampled. Roots with adhering soil (RAS) were carefully separated from bulk sand and sand soil mix by shaking. Soil and root dry mass (RT) was recorded after drying the samples at 105°C, and the RAS/RT ratio was calculated. Root hair length and density were evaluated using 12 plants. Plantlets were carefully separated from soil by shaking. After the loose soil separation the roots of plantlets were washed in distilled water and left to dry in Petri dishes containing 5 ml of water. The other set of plants was homogenized and used for bacterial identification and quantification. Water use efficiency (WUE) was calculated as total dry mass/total water usage. Plant relative water content (RWC) after inoculation with A26 and A26Δ*sfp* was estimated as described elsewhere using the formula RWC (%) = [(W-DW)/TW-DW)] × 100. W is sample fresh weight, TW sample turgid weight and DW sample dry weight. Eight independent experiments were performed.

### Studies on the role of A26 Sfp-type PPTase mediated secondary metabolites

#### Culture vessel assay

To study A26 Sfp-type PPTase mediated metabolite direct effect on wheat root growth the metabolite extraction of A26 and A26Δ*sfp* was performed as described by Li et al. (2007). Fusaricidin and polymyxin bioactivity assays were conducted as described earlier (Choi et al., 2008, 2009). Active fractions were evaporated to dryness and taken with saline to initial culture volumes. Wheat seeds were germinated for 24 h, transformed to culture vessels in water for 48 h, immersed in A26 and A26Δ*sfp* metabolite extracts for 12 h at 25°C, rinsed briefly with water and placed in saline for a further 96 h. Then dry weights of the lipopeptide-treated roots were estimated and roots were prepared for scanning electron microscopy (SEM). To compare the metabolite effect with the effect of A26 and A26Δ*sfp* the bacterial cultures were grown in TSB o/n and diluted to 10^7^/ml. Wheat seeds were germinated for 24 h, transformed to culture vessels in water for 48 h, inoculated with A26 and A26Δ*sfp* cultures for 2 h at 25°C, rinsed briefly with water and placed in saline for a further 96 h. Dry weights of the lipopeptide-treated roots were estimated and roots were prepared for SEM.

#### Sand soil assay

The other set of A26, A26Δ*sfp* and their metabolite treated roots were placed on sand soil left to grow for 5 more days in controlled environment as described above. Soil moisture content was kept constant. Seven day old seedlings drought stress was induced by stopping watering.

#### Sand soil assay with polymyxin B or polymyxin E

Commercial antibiotics polymyxin B or polymyxin E sulfate salts (Sigma-Aldrich) were used. Seeds were treated with three concentrations of the antibiotics (0.3, 2.5, and 7.5 μg/ml) and left to grow in sand soil in controlled environment and constant soil moisture. Seven day old seedlings were exposed to drought stress by stopping watering. Dry weight experiments were performed after 6 days. Each value represents the mean of three experiments.

### Protein extraction and antioxidant enzyme activity measurements

Leaf samples for enzyme activity determination were taken after 8 days from drought-treated and well-watered plants. Plant tissue was mixed with 10 ml extraction buffer as described by Knöerzer et al. (1996). The mixture was centrifuged at 14,000 rpm (Eppendorf, 5415C) for 10 min at 5°C, and the supernatant was used to determine protein content and activity of key antioxidant enzymes. Monodehydroascorbate reductase (MDAR) activity was determined following the decrease in light absorbance at 340 nm due to NADH oxidation as described by Hossain et al. (1984). Glutathione reductase (GR) activity was determined by increase in absorbance at 412 nm according to Smith et al. (1989). Superoxide dismutase (SOD) activity was determined by reduction in light absorbance at 490 nm using an Oxiselect SOD activity assay kit (Cell Biolabs, San Diego, CA, USA) according to manufacturer's instructions. Catalase (CAT) activity was measured by reduction in light absorbance at 520 nm, using an OxiselectTM CAT activity assay kit (Cell Biolabs). For CAT and SOD, enzyme activities were determined per gram of fresh mass (FM). Ultimately, the enzyme activities were normalized with respect to the activities in well-watered plants. The corresponding activities were 20 nmol (mg FM)^−1^ min^−1^ for MDHAR, 25 nmol (mg FM)^−1^ min^−1^ for GR, 0.45 μmol (g FM)^−1^ min^−1^ for SOD and 53 μmol (mg FM)^−1^ for CAT.

### Biofilm formation assay

The assay was performed based on pellicle weights as described by Beauregard et al. (2013). Briefly, cells were cultured from 1 day old colonies resuspended in 3 ml potato dextrose broth (PDB). After 2 h the cells were diluted 1:100 in 3 ml PDB. The dilution was repeated two more times. After the last dilution, cells were harvested at *OD*_600_ < 0.5 and adjusted to a final *OD*_600_ of 0.3. The assays were performed in 24 well plates. Pre-weighed PELCO prep-eze individual wells with a mesh bottom (opening size 420 uM) (Ted Pella) were put in the wells to which 1 ml medium and 14 ul of cells were added. Plates were incubated at 30°C for 96 h to allow pellicles to develop. Individual wells were then removed, dried and weighed. Each value represents the mean of three experiments.

### B complementation assay with A26 and A26Δ*sfp* metabolite extracts

To study the Sfp-type PPTase mediated secondary metabolite effect on A26, A26Δ*sfp*, and A26Δ*sfp*/pHPS9-*sfp* biofilm formation the metabolite extraction was performed as described by Li et al. (2007). Fusaricidin and polymyxin bioactivity assays were conducted as described earlier (Choi et al., 2008, 2009). Active fractions were evaporated to dryness and taken with saline to initial culture volumes. Single colony selection plates were supplemented with the filter-sterilized metabolite extracts. Biofilm formation assays were performed as described above with exception that all growth media were supplemented with A26 or A26Δ*sfp* filter-sterilized metabolite extracts.

### Microscopy

Wheat seedlings were inoculated as described above with A26, A26Δ*sfp*, and A26Δ*sfp*/pHPS9-*sfp*. Another set of roots was prepared as described under culture vessel assay. Scanning electron microscopy (SEM) was performed as described earlier by Timmusk et al. (2005). Briefly, 10 day old plant root tips were fixed in glutaraldehyde or in a solution containing formaldehyde. The samples were dehydrated using a graded ethanol series and critical point dried in CO_2_. The pressure was decreased very slowly to prevent tissue damage. Samples were mounted on stubs and shadowed with gold (22 nm) before they were viewed with a Philips Autoscan SEM. All images were computer processed.

For the plant root hair assay, wheat seedlings were inoculated as described above with A26 A26Δ*sfp*, and A26Δ*sfp*/pHPS9-*sfp*. Ten day old plantlets were carefully separated from soil by shaking, and roots were washed in distilled water and left to dry in Petri dishes containing 5 ml of water. The dried root system was evaluated using a Zeiss LSM 710 microscope.

### Data confirmation and validation

Experiments were repeated three times to confirm reproducibility. Replicated data were analyzed by ANOVA, and all treatment effects were considered significant at a conservative level of significance of *P* ≤ 0.01.

## Results

### Bioinformatic analysis of the A26 Sfp-type PPTase gene

The analysis of A26 genome shows that it contains a single Sfp-type PPTase encoding gene *(ppa26_1634)*. The gene shares 97, 92 and 91% homology with *P. polymyxa* E681, SC2 and M1 PPTase genes, respectively and contains three conserved sequence motives described by Lambalot et al. (1996).

### Inactivation of A26 Sfp-type PPTase results in loss of non-ribosomal peptide production

**Figure 2:**
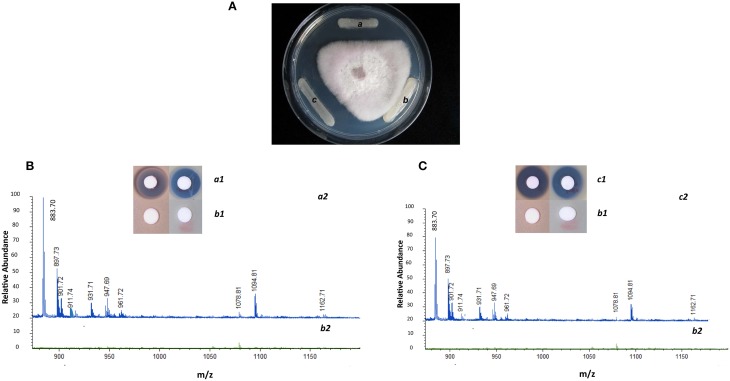
**Phenotypic and chemical analysis of *P. polymyxa* A26 Sfp-type PPTase mutant (A26Δ*sfp*). (A)** Inhibitory effect of wild type A26 (a), A26Δ*sfp* (b) and complemented strain A26Δ*sfp*/pHPS9-*sfp* (c) against *F. graminearum*. Note that the zone of antagonism observed with wild type has disappeared with mutant and is fully restored with complemented strain. **(B)** Antimicrobial activities of fusaricidin, polymyxin and MALDI-TOF MS analysis of culture extracts of wild type A26 (*a1* and *a2*, respectively; blue line) and A26Δ*sfp* (*b1* and *b2*; green line). Note that the fusaricidins (molecular weights 883, 897, 911, 931, 947, and 961 Da) and polymyxin (molecular weight 1094 Da) produced in wild type are eradicated in A26Δ*sfp* culture extracts. **(C)** Antimicrobial activities of fusaricidin, polymyxin and MALDI-TOF MS analysis of complemented strain A26Δ*sfp*/pHPS9-*sfp* culture extracts (*c1* and *c2*; blue line) is compared to mutant A26Δ*sfp* (*b1* and *b2*; green line). Note that synthesis of fusaricidins and polymyxin is fully restored by A26Δ*sfp* strain complemented with plasmid pHPS9-*sfp*.

The suicide vector was constructed as described on Figure 1. The initial gene replacement was performed in the *P. polymyxa* E681 strain. The chromosomal DNA was isolated from E681 and transformed into A26. After the confirmations by PCR and sequencing, the culture extracts were isolated from wild type strain, mutant strain and complemented strain. To date the only characterized non-ribosomal peptides in *P. polymyxa* are fusaricidins and polymyxins. As the *P. polymyxa* A26 sequence shares 96% homology with the well-studied *P. polymyxa* E681 sequence, comparative *in silico* prediction of *P. polymyxa* A26 NRPS based on E681 data and peptide structures according to NRPS architecture was performed using NRPS prediction program, ClustalW and MegAlign (Choi et al., 2008, 2009; Royer et al., 2013). The analysis confirms our LC-MS data and suggests that A26 is able to produce fusaricidins of molecular weights 883, 897, 911, 931, 947, and 961 Da and a polymyxin of molecular weight 1094 Da (Figure 2 and Figure S1). The extracts from the wild type, A26Δ*sfp* and complemented strain cultures were isolated, tested for bioactivity and analyzed by MALDI-TOF MS. Neither of the non-ribosomal peptides was detected in A26Δ*sfp* extracts (Figure 2). To prove that the phenotypic changes were due to Sfp-type PPTase replacement, the gene was cloned into the pHPS9 vector and transformed into A26 to complement the inactivated gene. In the complemented strain the wild type phenotype was fully restored (Figure 2). The mutant, wild type and complemented strains were tested on agar plates against a pathogen *F. graminearum*. A typical example is shown in Figure 2A.

### Inactivation of A26 Sfp-type PPTase results in greatly improved biofilm production

**Figure 3:**
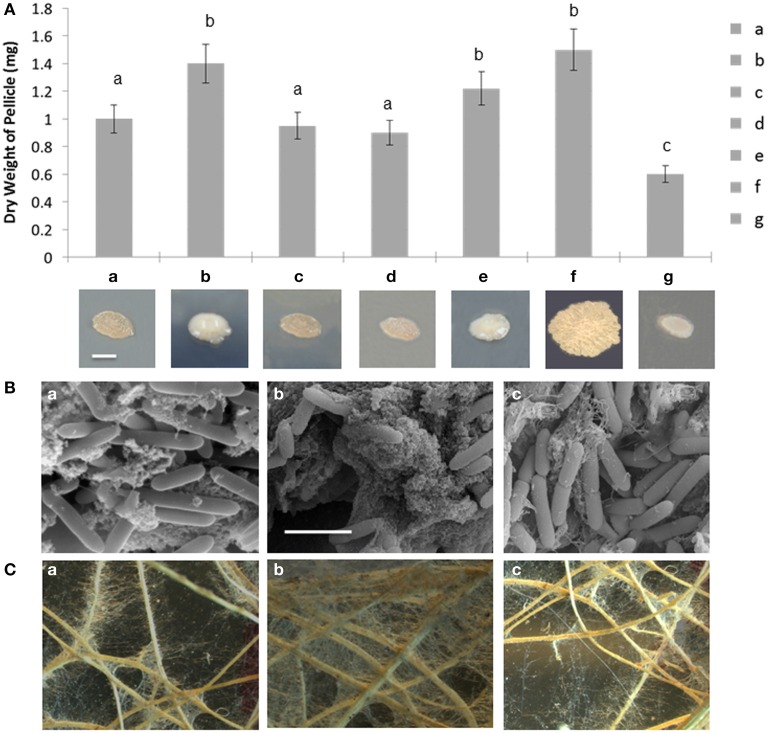
**Biofilm and root hair formation analysis of *P. polymyxa* Sfp-type PPTase mutants. (A)** *In vitro* biofilm formation of A26 (a), A26Δ*sfp* (b), A26Δ*sfp*/pHPS9-*sfp* (c), E681 (d), E681Δ*sfp* (e), compared to *B. subtilis* 3610 (f), and *3610*Δsfp (g). Colony phenotypes of the strains are shown. Colonies were grown on PDA agar for 4 days at 30°C. The scale bar represents 2 mm. **(B)** Scanning electron microscopic images of A26 (a), A26Δ*sfp* (b), A26Δ*sfp*/pHPS9-*sfp* (c) inoculated wheat roots. Significantly more biofilm compared to A26 is formed on the roots inoculated with A26Δ*sfp*; complementation of the strain with pHPS9-*sfp* restores the wild type biofilm formation level. The scale bar represents 3. **(C)** Light microscopic images of biofilm and root hair formation on wheat roots inoculated A26 (a), A26Δ*sfp* (b), and A26Δ*sfp*/pHPS9-*sfp* (c). Note that compared to A26 significantly more root hair and biofilm are formed on wheat roots inoculated with A26Δ*sfp*. Complementation of A26Δ*sfp* restores the wild type of root hair and biofilm formation levels.

Various assays were used to evaluate biofilm formation of the wild type, A26Δ*sfp* and complemented strain. Enhanced slime production and changed colony morphology were observed on plate assays PDA agar plates (Figure 3A). The deletion of the Sfp-type PPTase gene resulted in about 40% higher biofilm formation based on pellicle weight assay (Figure 3A). Complementation restored the biofilm formation rates to wild type level (Figure 3A). A26 and the other *P. polymyxa* strain E681 Sfp-type PPTase mutants were almost identical in their colony morphology but in E681Δ*sfp* the enhancement of biofilm formation (30–35%) was slightly smaller than in A26Δ*sfp* (40%). For comparison *B. subtilis* 3610 and its Sfp mutant were also studied for their biofilm formation. In contrast to *P. polymyxa* strains, *B. subtilis* 3610 Sfp mutation significantly impaired its colony morphology and biofilm formation. *B. subtilis* Sfp mutant impaired its pellicle weight by 60% (Figure 3A). SEM images of wheat root tips colonized with wild type, A26Δ*sfp* and complemented strain confirmed the results observed on colony morphology plate assays and biofilm formation pellicle assays. Significantly more porous extracellular matrix is formed by A26Δ*sfp* (Figure 3B). Complementation successfully restores the extracellular matrix formation to the wild type level (Figure 3B).

In order to study if the A26 Sfp-type PPTase mediated NRPs/PKs are directly involved in the observed biofilm promotion, additional weight-based biofilm formation assays were performed. Metabolite extracts from A26 and A26Δ*sfp* were exogenously added to A26, A26Δ*sfp* and its complemented strain A26Δ*sfp* pHPS9-*sfp* (**Table 2**). A26 Sfp-type PPTase mutant biofilm formation level was restored with the external addition of the A26 metabolites (**Table 2**). At the same time there was no significant effect of any other metabolite extract treatment on any tested strain (**Table 2**).

Another assay was performed with the A26 and A26Δ*sfp* inoculated plant roots grown in sand, washed and left to dry in 5 ml water on Petri plates. Biofilm formation was observed to significantly enhance root hair growth of A26Δ*sfp* inoculated plants (Figure 3C). Additional quantitative estimation of biofilm formation was performed based on amount of soil attached to roots (Table 1). Two times more soil was attached to the wheat seedling roots inoculated with A26Δ*sfp* (Table 1).

**Table 1:** **Comparative effect of priming by *P. polymyxa* A26 and A26Δ*sfp* on wheat *(Triticum aestivum L*. cv. *Stava)* average (±SD) growth characteristics, water use efficiency and antioxidant enzyme activities**.

	**Well-watered**		**Drought-stressed**	
	**A26**	**A26Δ*sfp***	**A26**	**A26Δ*sfp***
Average plant survival improvement (%)			200	600
Total lateral root length (cm)^1^	261 ± 12^a^^*^	284 ± 12^a^	84 ± 10^b^	141 ± 32^c^
Soil attached to root^2^ (g g^−1^ dry root)	61 ± 12^a^	91 ± 31^a^	11 ± 3^b^	23 ± 12^c^
Average root hair length^3^ (mm)	0.72 ± 0.23^a^	1.2 ± 0.2^b^	0.82 ± 0.22^a^	1.92 ± 0.21^c^
Root hair density (number mm^−3^)	22.1 ± 3.0^a^	30.2 ± 2.3^b^	24.3 ± 2.4^a^	32.2 ± 1.3^b^
Number of fresh roots per plant			3.2 ± 1.3^a^	10.2 ± 2.2^b^
Water use efficiency (g g^−1^)^4^	0.1049 ± 0.0029^a^	0. 1085 ± 0.0039^a^	0.0712 ± 0.0030^b^	0.1380 ± 0.0012^c^
Relative enzyme activities: ^5^				
MDHAR	1 ± 0.1^a^	1.3 ± 0.2^a^	1.7 ± 0.1^b^	2.5 ± 0.2^c^
GR	1 ± 0.2^a^	1.2 ± 0.1^a^	1.3 ± 0.3^a^	1.7 ± 0.1^b^
SOD	1 ± 0.1^a^	1.3 ± 0.1^b^	0.7 ± 0.1^c^	1.9 ± 0.1^d^
CAT	1 ± 0.1^a^	1 ± 0.2^a^	1.4 ± 0.1^b^	2 ± 0.1^c^

1 *Analysis of plant root was conducted by Root Reader3D Imaging and Analysis System and manually*.

2 *Twelve plants per treatment were sampled. Roots with adhering soil (RAS) were carefully separated from bulk soil by shaking. Soil and root dry mass (RT) was recorded after drying the samples at 105°C, and RAS/RT ratio was calculated*.

3 *Twelve plants were carefully separated from soil by shaking followed by washing the roots in distilled water and left to drain in Petri dishes with water to maintain humidity. Root system characteristics were evaluated by Zeiss LSM 710 microscope*.

4 *Water use efficiency is defined as the ratio of total plant dry mass per total water used*.

5 *MDHAR, Monodehydroascorbate reductase; GR, Glutathione reductase; SOD, Superoxide dismutase; CAT, Catalase. See SI Methods for enzyme extraction and activity measurements*.

* Values (mean value ± SE) followed by the same letter are not significantly

*different (*P* ≤ 0.01)*.

The metabolism of the wild type, mutated and complemented strains of *P. polymyxa* was studied for the species characteristic metabolic traits using the BioMerieux API50CH system. Similar metabolic traits were confirmed of all three strains. Additional biochemical identification tests were performed using the BioMerieux API20E system. Acetoin production was confirmed to be produced by the wild-type, mutant and complemented strain.

### Inactivation of A26 Sfp-type PPTase results in improved plant growth characteristics and drought tolerance enhancement

Comparative effects of the wild type A26 and A26Δ*sfp* on wheat (*Triticum aestivum* cv. Stava) growth characteristics water use efficiency and relative water content were studied. Initially the colonization ability of the strains was evaluated using PCR assays. The mutation did not influence the A26 root colonization and 10^5^ - 10^6^ cells of wild type as well as mutant were detected in plant roots. The mutant significantly increases seed germination, root hair length, density, amount of soil attached to roots and plant water use efficiency (Table 1). One hundred percent of the A26Δ*sfp*-primed seeds germinated under normal and stress conditions. A26Δ*sfp* increases root length and root hair length and density. A26Δ*sfp* inoculation resulted in 4.5 and 2.5 times improvements in root hair length and density, respectively. This is about twice the improvements obtained with the wild-type gene. The detailed analysis of plant root systems reveals that plant lateral root length was improved about 150 % by A26Δ*sfp* (Table 1). The inoculation with A26Δ*sfp* results in 1.6 times improved water use efficiency compared to the wild type and plant relative water content is significantly improved (Table 1 and Figure S2).

Antioxidant enzymes play important role in reactive oxygen species scavenging and plant drought tolerance enhancement. Hence the activities of monodehydroascorbate reductase (MDHAR), glutathione reductase (GR), catalase (CAT), and superoxide dismutase (SOD) were studied after 8 days in drought-stressed and well-watered plants. Enzyme activities were normalized to the well-watered seedlings enzyme activities. The relative activity of MDHAR was increased by A26 and A26Δ*sfp* colonization (Table 1). Activity of GR was slightly enhanced by A26, and significantly enhanced by A26Δ*sfp*. Both SOD and CAT activities were increased by A26Δ*sfp* under drought stress (Table 1).

**Figure 4:**
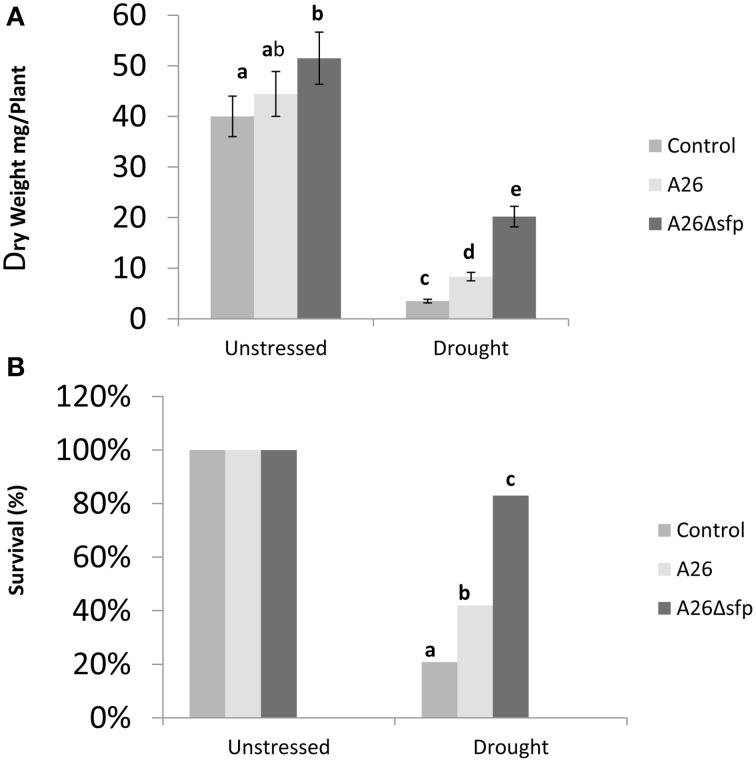
***P. polymyxa* A26 and its Sfp-type PPTase mutant (A26Δ*sfp*) wheat drought tolerance enhancement analysis**. Effect of A26 and A26Δ*sfp* inoculation on wheat dry mass **(A)** and survival **(B)**.

The comparative effects of inoculation with A26 or A26Δ*sfp* on the survival and dry weight of winter wheat (cv. Stava) plants grown in sand with 10% greenhouse soil and exposed to drought stress was investigated. Dry weights were estimated after 14 days and survival after 14 days of drought exposure and 4 days recovery watering. A26Δ*sfp* improved plant dry weight under drought stress about 6 times, which is 3 times improvement over wild type (Figure 4A). A26Δ*sfp* primed plant survival was 4 times higher than controls after 14 days of drought stress and 4 days of recovery watering (Figure 4B). This is twice the increase obtained with the wild type.

### A26 Sfp-type PPTase- mediated metabolites and polymyxin B and E induce negative effects on wheat seedlings

**Figure 5:**
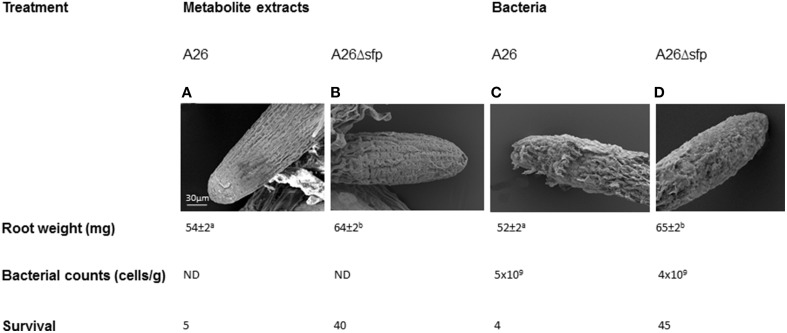
**Direct effect of A26 Sfp-type PPTase mediated metabolites on wheat root**. Scanning electron microscopic images, dry mass, root bacterial counts and survival of A26 **(A)**, A26Δ*sfp* metabolite extract treated roots compared to **(B)**, A26 **(C)**, and A26Δ*sfp* bacterial culture **(D)** treated roots.

Wild type A26 and A26Δ*sfp* bacteria and their metabolite extracts were studied for biological activity on wheat root growth in water vessels. A26 and its extracts caused damage and reduced root growth compared to A26Δ*sfp* bacteria and their metabolite extracts (Figure 5). Both bacterial root counts were 10^9^/cells per g root after 96 h incubation in saline. Wheat seedlings inoculated with A26 or A26Δ*sfp* or treated with the respective metabolite extracts, were grown in sand and exposed to drought stress. 4–5% of wild type culture or metabolite treated survived the 5 day stress (Figure 5). Forty to forty five percent of wheat seedling treated with A26 or A26Δ*sfp* bacteria or metabolites survived 5 days of drought stress (Figure 5).

**Figure 6:**
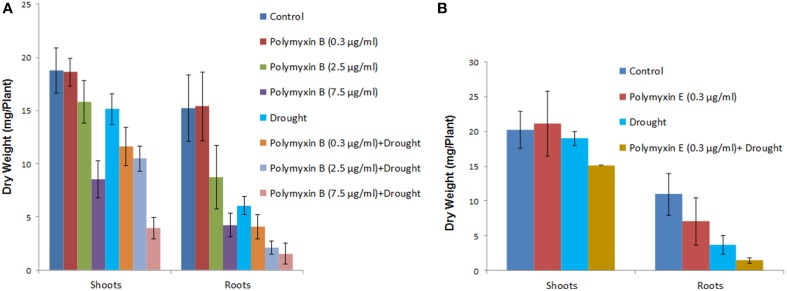
**Direct effect of polymyxin B and polymyxin E on wheat root**. Three concentrations of the antibiotics (0.3, 2.5 and 7.5 μg/ml) were used in the study. Effect of polymyxin B **(A)** and polymyxin E **(B)** on shoot and root dry weight under well watered regime and drought stress. Note that due to germination impairment by polymyxin E only lowest concentration of polymyxin E treated plant dry weights of shoots and roots are shown.

Three concentrations of the antibiotics polymyxin B and E (0.3, 2.5, and 7.5 μg/ml) were used to study direct effects of lipopeptides on the growth of wheat seedlings. Dry weights of the plants treated with polymyxin B and E at the two higher concentrations (2.5 and 7.5 μg/ml) were significantly reduced under both the well watered and under drought conditions (Figure 6). All concentrations of both antibiotics had significant negative effect on plants under drought stress (Figure 6). Owning to germination impairment by polymyxin E only lowest concentration of polymyxin E was studied for its effect on the dry weight of shoots and roots.

## Discussion

Here we report on the inactivation of the Sfp-type PPTase gene of *P. polymyxa* A26. Recently we published a simplified method for *P. polymyxa* genetic manipulation (Kim and Timmusk, 2013). Despite the success of direct manipulation of the well-studied laboratory strain E681, we were not able to carry out Sfp-type PPTase gene homologous recombination in the natural isolate A26. This further confirms the diverse nature of *P. polymyxa*. Some of the bacterial natural isolates are recalcitrant to any kind of genetic manipulation (Timmusk et al., 2005). Hence the initial gene replacement was performed in the *P. polymyxa* E681 strain (Figure 1). The chromosomal DNA was isolated from E681 and transformed into A26. Bioactivity and plate antagonism assays, and MALDI-TOF MS analysis, confirm the loss of fusaricidins and polymyxin production by the mutant and successful restoration by the complemented strain (Figure 2). The results indicate that, similar to non-ribosomal peptide/polyketide synthesis in various beneficial and pathogenic bacteria, *P. polymyxa* A26 is dependent on the presence of a single functional Sfp-type 4′-phosphopantetheinyl transferase identified in its genome (Nakano et al., 1988; Chen et al., 2009; Leblanc et al., 2012).

### Inactivation of A26 Sfp-type PPTase results in greatly improved biofilm production

The wild type, mutant and complemented strain metabolism was studied using BioMerieux® systems and no metabolic differences were detected. The major phenotypic difference of the mutant strain is its enhanced biofilm production (Figure 3 and Table 1). *B. subtilis* strain 3610, which is genetically almost identical to *B. subtilis* 168, was used in the study. It is generally known that the Sfp mutation impairs *Bacillus* spp. biofilm formation and for that reason root colonization is also impaired (Bais et al., 2004; Angelini et al., 2009; Chen et al., 2009; Dietel et al., 2013; Mielich-Suss and Lopez, 2014; Zeriouh et al., 2014). Using the weight-based quantification assay we showed that *B. subtilis* 3610 Sfp mutant biofilm formation was about 60% impaired. At the same time biofilm formation in the A26 Sfp-type PPTase mutant was increased about 40% (Figure 3). The A26Δ*sfp* biofilm formation enhancement is confirmed *in vitro* on plate assays and liquid assays as well as *in planta* on wheat roots using scanning electron and light microscopy and indirectly by soil attached to roots (Figure 3 and Table 1). In order to confirm the A26 biofilm promotion by the mutation the assays were performed with another isolate E681Δ*sfp*. A significant, but slightly smaller, increase in biofilm formation compared to A26Δ*sfp* was observed (Figure 3). The link between lipopeptide antibiotic production and biofilm formation was suggested by Li et al. who observed increased slime production of the *P. polymyxa* PKB1 lipopeptide mutant (Li et al., 2007).

### Mechanism of Sfp-type PPTase mediated biofilm formation

The biofilm increase of *P. polymyxa* Sfp-type PPTase mutants is in contrast to the *Bacillus* spp. model (Angelini et al., 2009; Lopez et al., 2009, 2010a,b; Mielich-Suss and Lopez, 2014; Zeriouh et al., 2014). The PPTase Sfp was first identified in 1988 by Nakamo (Nakano et al., 1988). Since then the enzyme has been studied in various organisms but *B. subtilis* is the organism where biofilm formation and related Sfp gene involvement is understood in molecular detail (Branda et al., 2001; Chu et al., 2008; Lopez et al., 2009, 2010a,b; McLoon et al., 2011; Beauregard et al., 2013; Vlamakis et al., 2013). In accordance with the model surfactin induces potassium leakage, which stimulates the activity of membrane protein kinase, kin C. Kin C activates the master regulator for biofilm formation. The stimulation may be triggered by a variety of natural products that cause cell membrane potassium leakage (Lopez et al., 2009). In contrast to *B. subtilis P. polymyxa* is a bacterium that is famously hard to manipulate. It is only during recent years that technologies have been discovered that somewhat simplify genetic manipulation of the species and allow mechanistic studies (Kim and Timmusk, 2013). Although *P. polymyxa* is one of the best rhizosphere biofilm formers, the mechanism of its biofilm formation has not been studied owning to difficulties to work with the species. An examination of the A26 genome indicates that no surfactins are encoded, whereas genes coding for polymyxins, fusaricidins as well as quite a number of potentially new non-ribosomal lipopeptides/antibiotics are present. The enzymes, NRPS and PKS required for the synthesis, are post-translationally modified by a single Sfp-type PPTase. The A26 Sfp-type PPTase inactivation eliminates the secondary metabolites (Figure 2). In order to study if the compounds are directly involved in the observed biofilm promotion, additional weight-based biofilm formation assays were performed. Metabolite extracts from A26 and A26Δ*sfp* were exogenously added to A26, A26Δ*sfp* and its complemented strain (Table 2). A26 Sfp-type PPTase mutant biofilm formation level was restored with the A26 metabolite complementation and there was no effect with any other metabolite exogenous addition (Table 2). This confirms that the Sfp-type PPTase- mediated A26 secondary metabolites are directly involved in its biofilm formation. Studies are directed now to identify the key regions in the respective synthetase gene clusters, and perform knockouts of all known and potential lipopeptide candidates.

**Table 2:** ***In vitro* biofilm formation of A26, A26Δ*sfp*, and A26Δ*sfp* pHPS9-*sfp* complemented with A26 and A26Δ*sfp* metabolite extracts**.

**Strain**	**A26**			**A26Δ*sfp***			**A26Δ*sfp*/pHPS9-*sfp***		
**Complementation**	**Control**	**A26**	**A26Δ*sfp***	**Control**	**A26**	**A26Δ*sfp***	**Control**	**A26**	**A26Δ*sfp***
Pellicle dry weight (mg)	0.9 ± 0.2^a^^*^	0.8 ± 0.2^a^	0.9 ± 0.1^a^	1.5 ± 0.2^b^	1.0 ± 0.1^a^	1.4 ± 0.1^b^	1.0 ± 0.1^a^	1.0 ± 0.1^a^	0.9 ± 0.1^a^

* *Values (mean value ± SE) followed by the same letter are not significantly different (*P* ≤ 0.01)*.

### Inactivation of A26 Sfp-type PPTase results in wheat drought tolerance enhancement

Our studies show that the A26 Sfp-type PPTase deficient mutant greatly enhances wheat drought tolerance (Figure 4). The increase is confirmed by survival, dry mass, relative water content water use efficiency and antioxidant enzyme analyses (Figure 4, Figure S2, and Table 1). The discovery opens new areas for biotechnology in the changing world as wheat is the primary staple food of mankind. It is generally believed that bacterial capacity to form biofilms on the root is required for the strain colonization and beneficial effect (Bais et al., 2004; Timmusk et al., 2005, 2009, 2014; Haggag and Timmusk, 2008; Timmusk and Nevo, 2011; Chen et al., 2013; Dietel et al., 2013; Garcia-Gutierrez et al., 2013; Zeriouh et al., 2014). It is tempting to speculate that biofilm formation facilitates the A26Δ*sfp* colonization and that the drought tolerance enhancement is linked to the improved colonization. However, we showed that the colonization by the wild type mutant and complemented mutant did not differ significantly. Can plants benefit from a bacterial biofilm? Bacterial biofilms are comprised of cells and extracellular matrix and form layers around a root hair (Figure 3). The dense biofilm matrix limits diffusion of ACC deaminase and biologically active compounds secreted by bacteria, and these are therefore concentrated for plant uptake (Table 1). It is suggested that *B. subtilis* biofilm may be generated in response to antimicrobials produced by other microorganisms and may thus constitute a defense mechanism to protect *B. subtilis* from the action of antibiotics in natural settings (Lopez et al., 2009). *P. polymyxa* is generally considered as a great biofilm forming biocontrol agent which owning to its unique antibiotic spectra is even able to form single species root biofilms under natural conditions (Timmusk et al., 2005; Timmusk and Nevo, 2011; Timmusk et al., 2011). Hence, A26Δ*sfp* enhanced tight extracellular matrix around roots may contribute to observed drought tolerance enhancement without being its primary cause.

### A26 Sfp-type PPTase mediated NRP/PK compounds induce negative effects in wheat seedlings and affect plant drought tolerance

A26 and its Sfp-type PPTase mutant metabolism was studied using BioMerieux® system. No metabolic differences were detected between the strains. It is confirmed in our MALDI-TOF MS that no lipopeptide antibiotics were produced by A26Δ*sfp* in contrast to A26 (Figure 2). Is there any direct effect of *P. polymyxa* Sfp-type PPTase mediated secondary metabolites on the host plant? To study the effect under gnotobiotic conditions, germinated wheat seeds were exposed to high inoculum densities of A26, A26Δ*sfp* or their lipopeptide extracts and synthetic polymyxins. A26 and its extracts reduced root growth, caused damage to the root tip and synthetic polymyxins had negative effect on seedlings drought tolerance (Figures 5, 6). Lipopeptides, which are amphiphilic molecules with an amino or hydroxy-fatty acid integrated into a peptide moiety, interact with the biological membranes and at some concentrations may induce cell leakage and death (Ongena et al., 2007; Zeriouh et al., 2011). We have previously reported that plant growth promoting *P. polymyxa* strains may cause mild negative effects on plant root tips which induce plant systemic resistance against *Erwinia carotovora* (Timmusk and Wagner, 1999; Timmusk et al., 2005). Formerly it has been suggested that microbial hydrolytic enzymes and auxins may be responsible for the deleterious effects (Timmusk and Wagner, 1999; Timmusk et al., 2005; Ludwig-Muller, 2015). The results here however support that NRP/PK-origin compounds produced by *P. polymyxa* may be the primary reason for its temporary mild deleterious influence (Figure 5). Even though several studies have speculated on rhizobacterially produced lipopeptide role in plant induced systemic resistance, the first evidence was provided only in 2007 (Ongena et al., 2007; Ongena and Jacques, 2008).

*P. polymyxa* A26 which is isolated from SFS, EC in the northern part of Israel is known to be a biofilm-forming ACC deaminase-containing phosphorus-solubilizing biocontrol bacterium capable of moderate plant drought tolerance enhancement (Timmusk et al., 2011, 2014). There are certainly more scenarios behind A26Δ*sfp* enhanced drought tolerance but our results indicate that the bacterial Sfp-type PPTase- mediated compound restriction contributes to the observed drought tolerance enhancement. The high inoculum densities of *P. polymyxa* used in our gnotobiotic experiment (Figure 5) don't occur in natural settings (Timmusk et al., 2005). Yet we show that the bacterial lipopeptide production might be the reason for temporary negative effects which induce plant systemic resistance but affect negatively plant drought tolerance. Salicylic acid related defense pathway activation during the host plant interaction has been shown by various plant-beneficial microorganisms, indicating that temporary mild negative effects under particular environmental conditions may be widely spread among plant growth promoting microbes (Phi et al., 2010; Alonso-Ramirez et al., 2014). Lipopeptide antibiotics have been successfully employed in the treatment of various pathogens since 1950s. The bulk of the work has focused on classifying modes of their action. How much of the lipopeptides are produced by A26 under natural conditions? Which of its Sfp-type PPTase-mediated compounds are involved in direct antagonism and induced systemic resistance? It is clear that the physiological concentration of any biologically active compound varies considerably depending on the dose and on all manners of parameters of treated host. More studies need to be performed on the exciting area of the A26 lipopeptide production under natural conditions. Cell type specific transcriptional regulation probing should reveal exactly how wheat root cells are influenced by A26 and A26Δ*sfp* priming. An important challenge for the future will be to determine which of the Sfp-type PPTase- mediated compounds contribute to the A26Δ*sfp* strain drought tolerance enhancement and biofilm promotion.

Rhizobacterial ability to enhance plant drought stress tolerance was serendipitously discovered in 1999 by Timmusk and Wagner in an attempt to study the soil bacteria for plant induced resistance and nitrogen fixation ability (Timmusk and Wagner, 1999). Recently the discovery was updated with an in principle new technology showing that the bacteria from harsh environments are more likely to be efficient in enhancing host plant drought tolerance (Timmusk et al., 2014). Here we show that the Sfp-type PPTase gene in *P. polymyxa* is a gate-keeper for the bacterial drought tolerance enhancement.

### Conflict of interest statement

The authors declare that the research was conducted in the absence of any commercial or financial relationships that could be construed as a potential conflict of interest.

## Supplementary material

Table S1 **Primers used in this study**.

Figure S1 **Analysis of *P. polymyxa* A26 fusaricidins synthesis. (A)** LC analysis of A26 culture supernatants. **(B)** MS data for the fusaricidins produced by A26.

Figure S2 **Relative water content (RWC) of *P. polymyxa* A26Δ*sfp*, A26 and untreated wheat under drought and well watered regime**.
