# Supplementary material for: Sfp-type PPTase inactivation promotes bacterial biofilm formation and ability to enhance wheat drought tolerance
Source: Front Microbiol. 2015 May 21;6:387. doi: 10.3389/fmicb.2015.00387 (PMC4439574; doi:10.3389/fmicb.2015.00387)
Supplement: Table S1 — Primers used in this study. [file Table1.DOCX]

**Table S1. Primers used in this study**

| sfp FF | 5’-CTC ATG CAT CAT TGT AAA TCA CTT TCG GAC G-3’ |
| --- | --- |
| sfp FR | 5’-CTC GGA TCC TCT TAA CAG CAC ATC GGC AT-3’ |
| sfp RF | 5’-CTC TCT AGA GAA GTC TTT TTC ATT CGA GCT-3’ |
| sfp RR | 5’-CTC GGG CCC TAA TCC GTT CAA GCG TCC AT-3’ |
| Cn F | 5’- GTG ACA AGG GTG AAA CTC-3’ |
| A26 sfp LF | 5’- CTC GGA TCC AGC ATA ATA CAC AGC AAG CGG G-3’ |
| A26 sfp LR | 5’- CTC GAA TTC CCT TAC CGG TCA ACT GCA AT-3’ |
| 1492 R | 5’-GGTTACCTTGTTACGACTT-3’ |
| 27 F | 5’-AGAGTTTGATCCTGGCTCAG-3’ |
