# Supplementary figures and images for: Sfp-type PPTase inactivation promotes bacterial biofilm formation and ability to enhance wheat drought tolerance
Source: Front Microbiol. 2015 May 21;6:387. doi: 10.3389/fmicb.2015.00387 (PMC4439574; doi:10.3389/fmicb.2015.00387)

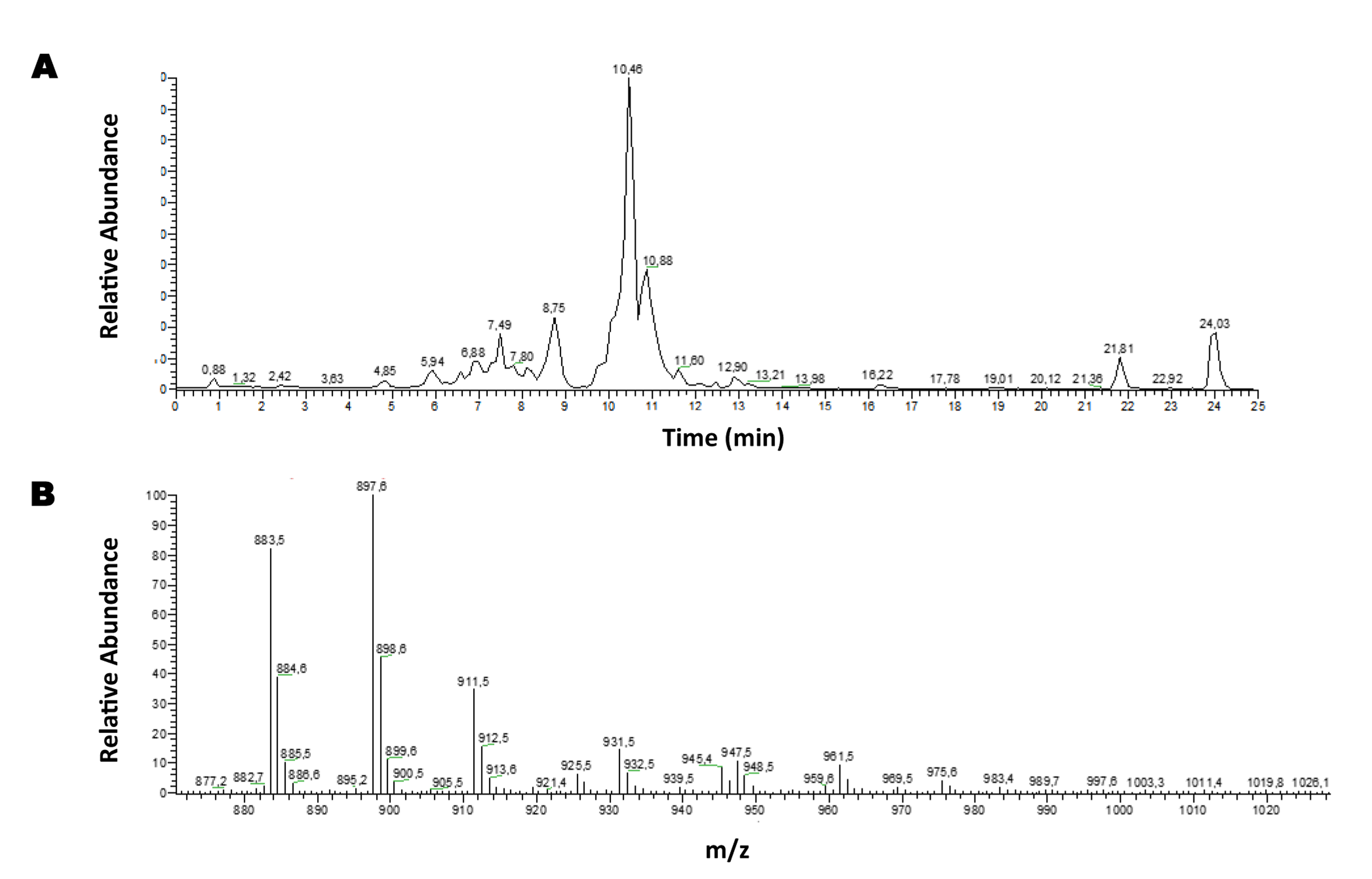

Supplement: Figure S1 — Analysis of P. polymyxa A26 fusaricidins synthesis. (A) LC analysis of A26 culture supernatants. (B) MS data for the fusaricidins produced by A26. [file Image1.TIF]

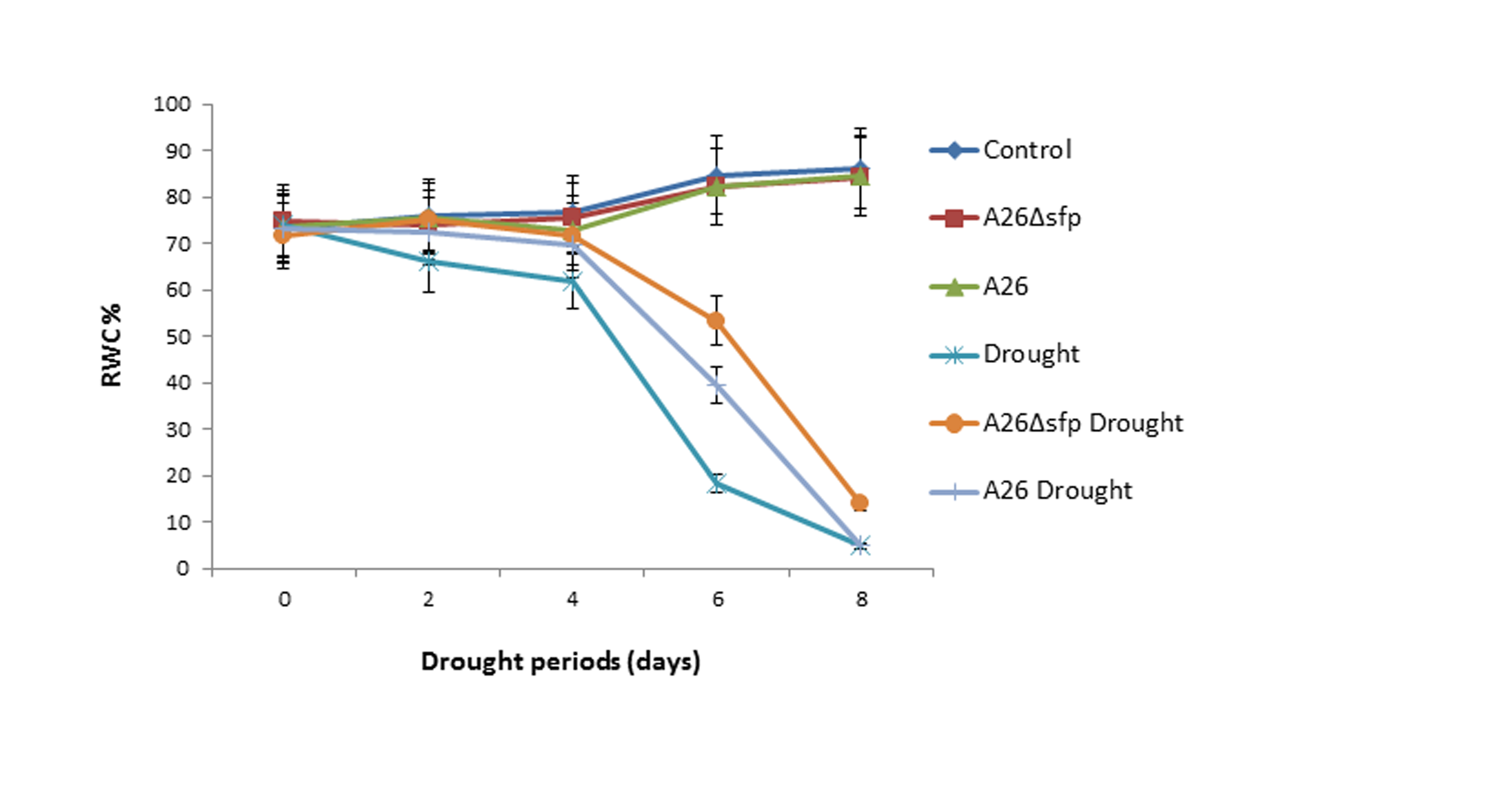

Supplement: Figure S2 — Relative water content (RWC) of P. polymyxa A26Δsfp, A26 and untreated wheat under drought and well watered regime. [file Image2.TIF]
